# Synthesis and Electrochemical Performance of Molybdenum Disulfide-Reduced Graphene Oxide-Polyaniline Ternary Composites for Supercapacitors

**DOI:** 10.3389/fchem.2018.00218

**Published:** 2018-06-12

**Authors:** Li-Zhong Bai, Yan-Hui Wang, Shuai-Shuai Cheng, Fang Li, Zhi-Yi Zhang, Ya-Qing Liu

**Affiliations:** Shanxi Province Key Laboratory of Functional Nanocomposite Materials, North University of China, Taiyuan, China

**Keywords:** molybdenum disulfide, reduced graphene oxide, polyaniline, ternary composites, supercapacitors

## Abstract

Molybdenum disulfide/reduced graphene oxide/polyaniline ternary composites (MoS_2_/rGO/PANI) were designed and synthesized by a facile two-step approach including hydrothermal and *in situ* polymerization process. The MoS_2_/rGO/PANI composites presented an interconnected 3D network architecture, in which PANI uniformly coated the outer surface of the MoS_2_/rGO binary composite. The MoS_2_/rGO/PANI composites with a weight percent of 80% (MGP-80) exhibits the best specific capacitance (570 F g^−1^ at 1 A g^−1^) and cycling stabilities (78.6% retained capacitance after 500 cycles at 1 A g^−1^). The excellent electrochemical capacitive performance is attributed to its 3D network structure and the synergistic effects among the three components that make the composites obtain both pseudocapacitance and double-layer capacitance.

## Introduction

To meet the burgeoning need of light-weight and portable electronic devices, efficient and environmentally-friendly electrochemical energy storage systems are urgently developed (Liu et al., 2017). Among the various energy storage systems, supercapacitors have drawn tremendous research attention due to their low cost, environmental friendliness, fast charging and discharging rate, excellent power density, high cycling stability, and long cycle life (Dunn et al., 2011; Yu et al., 2013; Wu et al., 2017; Qu et al., 2018). According to the charge-storage mechanism, supercapacitors are classified into two categories: electrochemical double-layer capacitors (EDLCs) and pseudocapacitors (Zhang et al., 2016). In general, pseudocapacitors of transition metal oxides and conducting polymers possess much higher specific capacitance than EDLCs of carbon materials, but their cycling stability is inferior (Li et al., 2014). Naturally, to draw on each other's strengths, a binary or ternary hybrid material composed carbon materials, a transition metal oxide and conducting polymer is more effective for high specific capacitance and long life time (Chen et al., 2014).

Graphene, a two-dimensional monolayer of sp^2^ carbon atoms, is considered as an extremely promising candidate for future advanced applications in supercapacitors due to its excellent electrical conductivity, high surface area, good mechanical flexibility, and chemical stability (Zhang et al., 2012). Meanwhile, it is an ideal substrate for the growth and anchoring of nanomaterials, such as metal oxide, metal sulfide and conducting polymers, to exploring hybrid composite for improved electrochemical properties (Zhao et al., 2018). Among various hybrid materials, graphene/MoS_2_, graphene/PANI, MoS_2_/PANI binary nanostructure are found to be promising electrode materials for supercapacitors (Ataca et al., 2012; Huang et al., 2013; da Silveira Firmiano et al., 2014; Thangappan et al., 2016). For example, Thangappan et al. reported a facile one step preparation of a molybdenum disulfide (MoS_2_) nanosheet-graphene (MoS_2_/G) composite with the *in situ* reduction of graphene oxide, which exhibited a high specific capacitance of 270 and 90 F g^−1^ at 0.1 and 1.0 A g^−1^, respectively. In addition, its specific capacitance can still remain 89.6% after 1,000 cycles at 0.6 Ag^−1^ (Thangappan et al., 2016). However, three major defects found in the binary composite including the low practical capacitance, poor cycle stability and poor rate performance, hinder its wide application in supercapacitors (David et al., 2014).

Recently, a ternary composite of MoS_2_/graphene wrapped with Fe_3_O_4_, polypyrrole and carbon nanotubes has been tried out in the fields of lithium ion batteries, electromagnetic wave absorption and electrocatalyst (Khan et al., 2016; Xie et al., 2016; Li et al., 2017). However, there are few reports on the synthesis of MoS_2_/rGO/PANI ternary nanostructure for supercapacitors applications.

In this work, we prepared molybdenum disulfide/reduced graphene oxide/polyaniline (MoS_2_/rGO/PANI) ternary composites by a facile two-step method. In the first step, the MoS_2_ nanosheets are uniformly grown on the surface of the GO nanosheets through a hydrothermal process to produce a MoS_2_/rGO binary composite. In the second step, the MoS_2_/rGO/PANI ternary composites were synthesized by *in situ* polymerization of aniline on the out face of the MoS_2_/rGO binary composite. In the MoS_2_/rGO/PANI ternary composites, the weight percent of PANI can effectively improve their electrochemical performance when they serve as the electrode materials in supercapacitors. The results indicate that the MoS_2_/rGO/PANI ternary composites deliver a very high specific capacity and excellent cyclic stability compared with the MoS_2_/rGO binary composite.

## Experimental

### Synthesis of a MoS_2_/rGO binary composite

Graphene oxide (GO) was synthesized from natural graphite flakes by a modified Hummers method (Zhang et al., 2015). The MoS_2_/rGO binary composite was prepared by a facile hydrothermal method. Typically, GO (0.8 g), (NH_4_)_6_Mo_7_O_24_·4H_2_O (1.236 g), thiourea (4 g) and HCl solution (0.2 ml) were dispersed in deionized water (40 ml) and sonicated for 1 h to form a uniform suspension. The above mixed dispersion was transferred into a Teflon-line stainless steel autoclave (50 ml) and annealed at 220°C for 18 h. After cooling down, the black precipitate was collected by centrifugation, washed with deionized water and ethanol for several times, and dried at 80°C for 24 h.

### Synthesis of MoS_2_/rGO/PANI ternary composites

The MoS_2_/rGO/PANI ternary composites were synthesized by *in situ* polymerization in the presence of MoS_2_/rGO and aniline. Typically, a certain amount of MoS_2_/rGO and dodecyl benzenesulfonic acid (4.065 g) were dispersed into 50 ml deionized water with ultrasonic radiation. Aniline (1.16 g) and deionized water (75 ml) were poured into the above suspension and sonicated for 1 h. A solution of APS (0.5 M, 25 ml) was dropwise added to the above mixed dispersion and continually stirred at 0°C for 5 h and then at 20°C for 2 h. After that, the blackish green product was filtered and washed with acetone, and then dried in a vacuum oven at 60°C for 8 h. The resultant MoS_2_/rGO/PANI ternary composites were denoted as MGP-X, where X (*X* = 50, 60, 70 and 80) represents the weight percentage loading of PANI in the ternary composites.

### Material characterization

The morphology and microstructure of the samples were characterized by a S-4800 scanning electron microscope (SEM) and a JEOL 2010 field-emission transmission electron microscope (TEM). The crystalline structures of the samples were performed on a Rigaku D/Max-2500 X-ray diffractometer with Cu Kα radiation (λ = 0.1542 nm) in the 2θ range from 5° to 90°. Fourier transform infrared (FT-IR) spectra were recorded on a Bruker Optics TENSOR 27 spectrometer using KBr pellets in the wave-number range of 400–4,000 cm^−1^.

### Electrochemical measurements

The electrochemical performance of the samples used as electrode materials for supercapacitors were measured in a three-electrode system. The working electrodes were fabricated by mixing 80 wt% active materials, 10 wt% carbon black, and 10 wt% polytetrafluoroethylene (PTFE) solution. The mixture was pasted on stainless steel network (1 × 1 cm^2^) and dried at 80°C for 12 h in a vacuum oven. The mass of active materials loaded on the working electrodes were 4–5 mg. A platinum foil and a saturated calomel electrode (SCE) were used as the counter electrode and reference electrode, respectively, and 1 M H_2_SO_4_ aqueous solution was used as the electrolyte. Cyclic voltammetry (CV) tests were obtained at different scan rates (1, 2, 5, 10, 20, 50, and 100 mV s^−1^) within a potential window of 0–1.0 V vs. SCE. Electrochemical impedance spectroscopy (EIS) measurements were carried out in the frequency range from 0.01 Hz to 100 KHz with 5 mV AC voltage amplitude at open circuit potential. Galvanostatic charge-discharge (GCD) investigations were performed at various current density (1, 2 3, 4, and 5 A g^−1^) in a potential range of 0–1.0 V vs. SCE. The capacities of the samples were calculated based on the mass of the active materials.

## Results and discussion

### Structure and morphology of MoS_2_/rGO/PANI ternary composites

**Figure 1:**
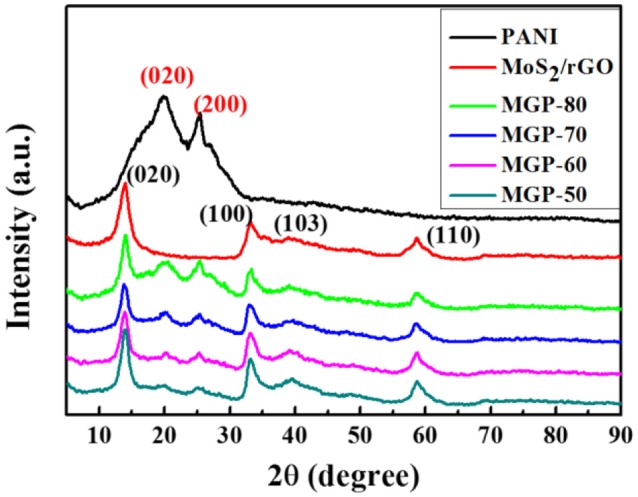
XRD patterns of the PANI, MoS_2_/rGO binary composite, and MoS_2_/rGO/PANI ternary composites.

Figure 1 shows XRD patterns of the PANI, MoS_2_/rGO binary composite, and MoS_2_/rGO/PANI ternary composites, respectively. The diffraction peaks of the PANI located at 2θ = 20.3° and 25.3°, which can be assigned to (020) and (200) crystal planes of the emeraldine PANI salt, respectively (Tong et al., 2014). The MoS_2_/rGO binary composite exhibits the diffraction peaks centered at 2θ = 14.1°, 33.1°, 39.6°, and 58.9°, which can be ascribable to the (002), (100), (103) and (110) crystal planes of 2H-phase MoS_2_ (JCPDS no. 37-1492), respectively. The diffraction peak of the rGO located at 2θ = 26.5° cannot be detected in the MoS_2_/rGO binary composite, which indicates that the restacking of graphene layers was inhibited by MoS_2_ nanosheets (Wang et al., 2014; Dai et al., 2017). In the diffraction spectrum of the MoS_2_/rGO/PANI ternary composites, there are some characteristic diffraction peaks of both MoS_2_/rGO and PANI, revealing that the PANI is successfully attached onto the surface of the MoS_2_/rGO binary composite. Moreover, the intensities of the diffraction peaks from PANI gradually increase with the elevated weight percent of PANI. All the results indicate that these three components are fully compounded together.

**Figure 2:**
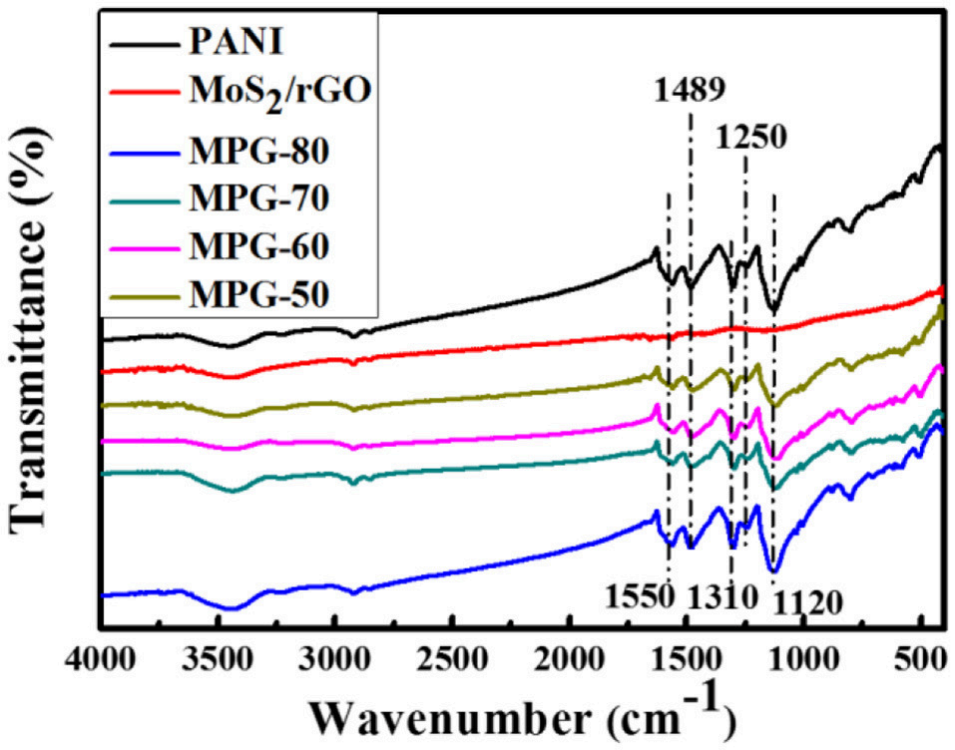
FT-IR spectra of the PANI, MoS_2_/rGO binary composite, and MoS_2_/rGO/PANI ternary composites.

Figure 2 shows FT-IR spectra of the PANI, MoS_2_/rGO binary composite, and MoS_2_/rGO/PANI ternary composites, respectively. As shown in Figure 2, the spectrum of PANI shows strong absorption peaks at 1,120, 1,250, 1,310, 1,489, and 1,550 cm^−1^ due to the C = N stretching, C–N stretching of the second amine, the aromatic C = C stretching vibration of the benzenoid and quinonoid rings, respectively (Cong et al., 2013). In the FT-IR spectra of the MoS_2_/rGO binary composite, there is no obvious peak arisen from the vibration of oxygen containing functional groups on GO, which is attributed to the reduction of graphene oxide after hydrothermal treatment. The weak peak at about 500 cm^−1^ is assigned to MoS_2_ vibration. Furthermore, the FT-IR spectra of MGP-50 show the obvious existence of all the PANI and MoS_2_/rGO characteristic peaks. With the increasing weight percent of PANI, the intensities of the main characteristic PANI peaks in the MoS_2_/rGO/PANI ternary composites all show an increase. This indicated that the PANI was successfully coated on the surface of the MoS_2_/rGO binary composite, which is helpful to improve the dispersibility of MoS_2_/rGO/PANI ternary composites in the electrolyte.

**Figure 3:**
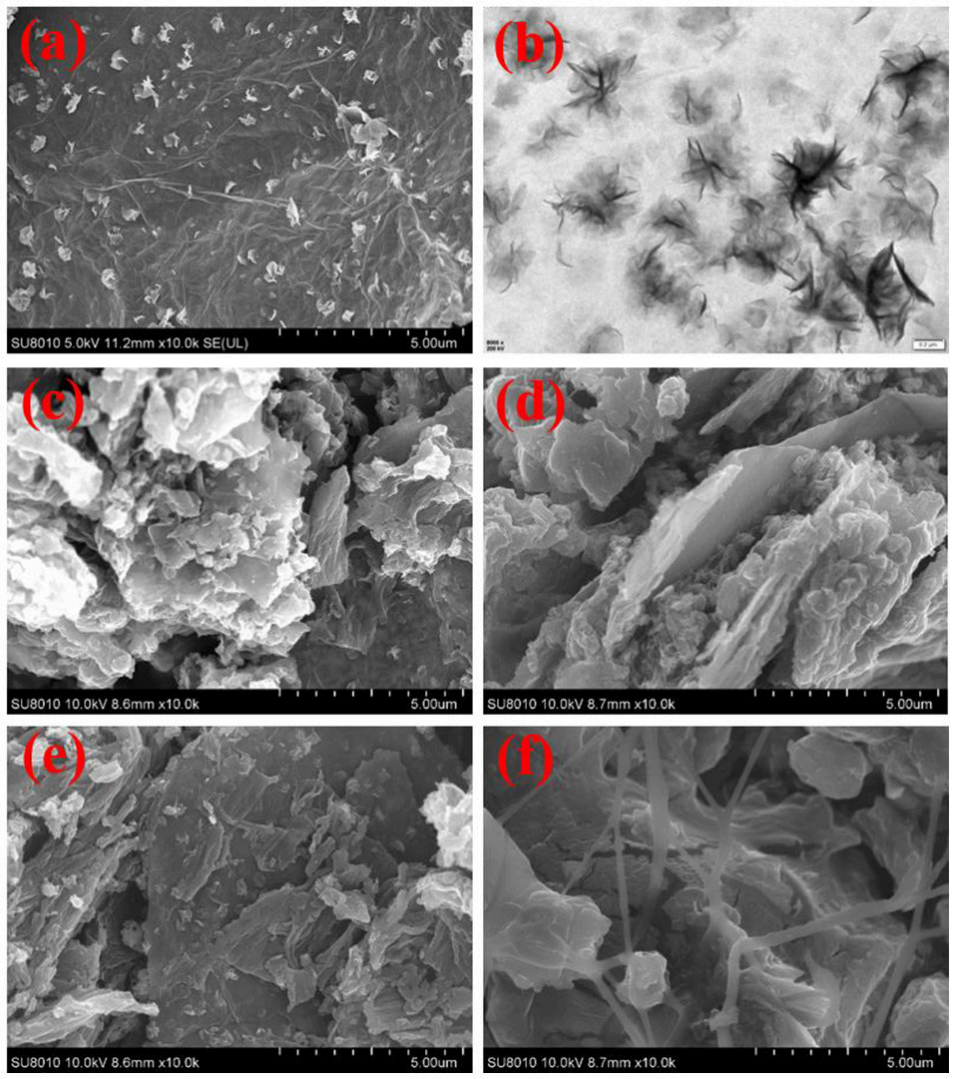
SEM and TEM images of the MoS_2_/rGO binary composite **(a,b)**, and SEM images of the MoS_2_/rGO/PANI ternary composites [MRP-50 **(c)**, MRP-60 **(d)**, MRP-70 **(e)**, and MRP-80 **(f)**].

The morphologies of the MoS_2_/rGO binary composite and MoS_2_/rGO/PANI ternary composites are shown in Figure 3. As shown in Figures 3a,b, the MoS_2_ nanosheets were well-scattered on the surface of rGO nanosheets to form hetero-layered architecture. The MoS_2_/rGO binary composite as an attractive substrate can supply a large number of active sites for the growth of PANI. Figures 3c–f show the morphology of the MoS_2_/rGO/PANI ternary composites with different weight percents of PANI. As shown in Figure 3c, the MGP-50 ternary composite shows that a well-defined and interconnected 3D network architecture. The PANI in a planar shape were polymerized and attached onto the surface of the MoS_2_/rGO binary composite due to the electrostatic interaction between negatively charged MoS_2_/rGO and positively charged PANI (Luo et al., 2015). With the increased weight percent of PANI, PANI coated MoS_2_/rGO assemble to generate a compact and laminated morphology so that the layer structure of MoS_2_/rGO is not clearly seen due to the low content (Figures 3d–f). Such particular structure of the MoS_2_/rGO/PANI ternary composites could increase the dispersion of PANI and improve the interfaces of PANI with electrolyte, which might be beneficial for the improvement of electrochemical performance of the MoS_2_/rGO/PANI ternary composite as electrode materials.

### Electrochemical performance of MoS_2_/rGO/PANI ternary composites

**Figure 4:**
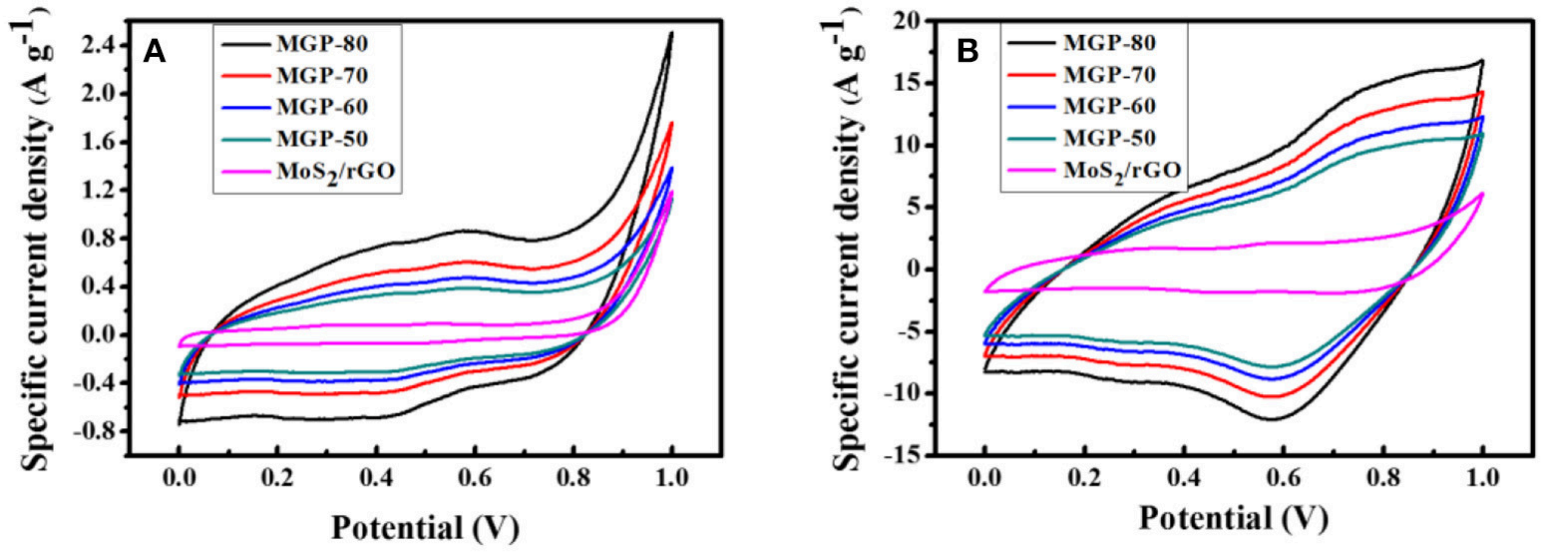
CV curves for the MoS_2_/rGO binary composite and MoS_2_/rGO/PANI ternary composites at scan rates of **(A)** 2 and **(B)** 50 mV s^−1^.

Figure 4 shows the CV curves of the MoS_2_/rGO binary composite and MoS_2_/rGO/PANI ternary composites at scan rates of 2 and 5 mV s^−1^. In the case of the MoS_2_/rGO/PANI ternary composites, it can be found that CV loop exhibit larger areas under redox curve than the MoS_2_/rGO binary composite, which indicates its higher specific capacitance. From the CV curves for the MoS_2_/rGO/PANI ternary composites at different PANI content, it was noted that the MGP-80 electrode shows the largest rectangular curve corresponding to the highest capacitance among the four samples. The CV curves for the MGP80 at a low scan rate of 2 mV s^−1^ show severely distorted rectangular shapes as well as two conspicuous pairs of small, broad oxidation and reduction peaks, resulting from the co-contribution of electrical double-layer capacitance generated from MoS_2_/rGO and pseudocapacitance arising from PANI (Wang et al., 2015). The redox peaks become less obvious, and the potential of the oxidation peak moves to higher potential while the potential of the reduction peak shifts to lower at a high scan rate of 50 mV s^−1^. It should be noted that MoS_2_/rGO/PANI ternary composites have fast ionic transport for charge-discharge operations, which results from synergetic effect of MoS_2_/rGO and PANI.

**Figure 5:**
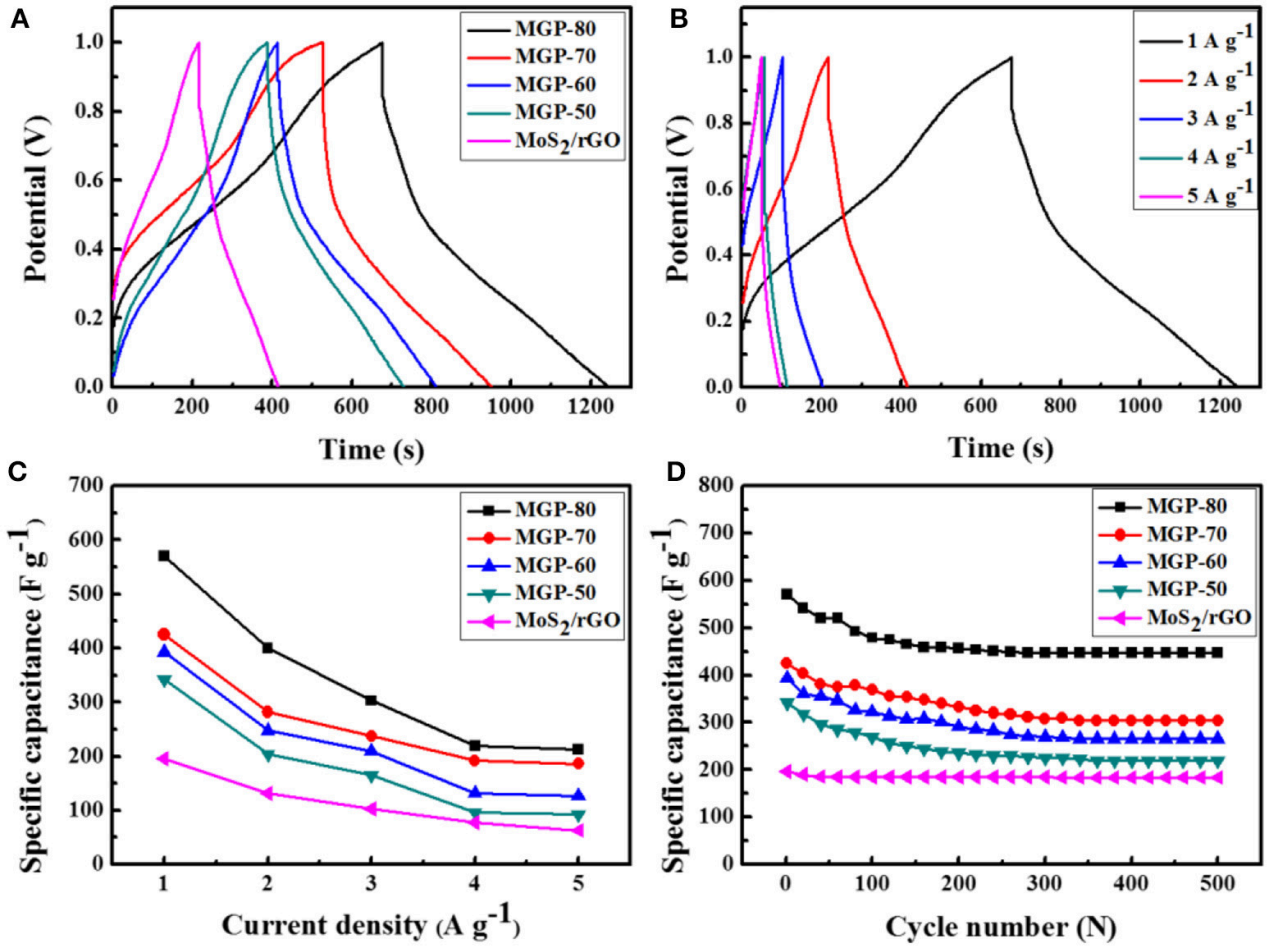
**(A)** GCD curves of MoS_2_/rGO binary composite and MoS_2_/rGO/PANI ternary composites at a current density of 1 A g^−1^, **(B)** GCD curves of the MGP-80 at different current densities, **(C)** the specific capacitance as a function of discharge current density; **(D)** cycle performance of the MoS_2_ /rGO/PANI ternary composites at 1 A g^−1^.

The supercapacitance behavior of the MoS_2_/rGO/PANI ternary composites was further investigated using GCD measurements, as shown in Figure 5. Figure 5A displays the GDC curves of the MoS_2_/rGO,MoS_2_/rGO/PANI with different amounts of PANI at a current density of 1 Ag^−1^. It can be seen that the shapes of the GDC curves of ternary composites are similar to that of binary composite, which indicates that the ternary composite possesses the co-contribution of electrical double-layer capacitance and pseudo-capacitance. The ternary composites with different amounts of PANI exhibit a higher discharge time and lower IR drop than binary composite, which indicates that the introduction of PANI can enhance the capacitance and conductivity (Wang et al., 2013). The discharge time of the ternary composites increased with an increase amounts of PANI. The MGP-80 ternary composite exhibits the maximum discharge time. Furthermore, the GCD curves of the MRP-80 at different current densities from 1 to 5 A g^−1^ are shown in Figure 5B. It could be seen that with the increase of current density, the discharge time of the composite decreased due to partial accessibility for electrolyte ions within the active material at high currents. Figure 5C reveals the specific capacitances of the ternary composites at the current densities of 1, 2, 3, 4, and 5 Ag^−1^. As can be observed, the capacitances of the MGP-80 were 570, 400, 303, 220, and 212 Fg^−1^, respectively. Figure 5D shows the cycle stability of the ternary composite at a current density of 1 A g^−1^. After 500 charging and discharging cycles, the specific capacitances of MPG-50, MPG-60, MPG-70 and MPG-80 retained 64.3, 67.7, 71.5, and 78.6% of the value of the first cycle, respectively. The good cycling stability of MPG-80 is ascribed to the fact that the synergistic effect between the MoS_2_/rGO and PANI could relieve the volumetric shrinkage or swelling of PANI during the charge/discharge process (Dai et al., 2013).

**Figure 6:**
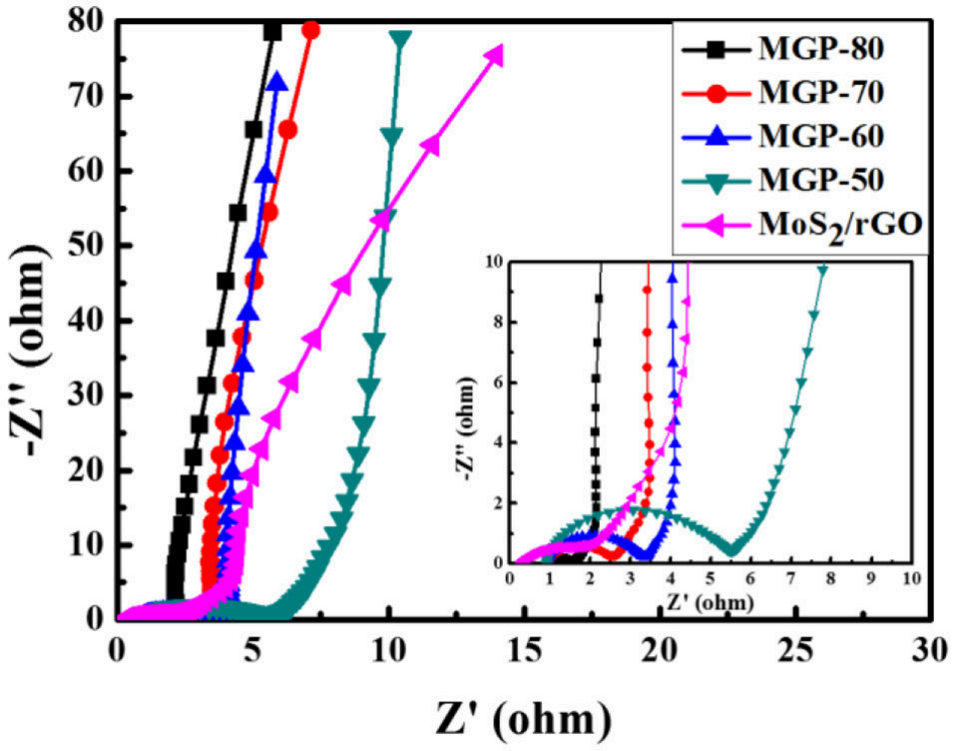
Nyquist plots of the MoS_2_/rGO binary composite and MoS_2_/rGO/PANI ternary composites (the inset is an enlarged view of the Nyquist curves).

EIS was carried out to describe the electrochemical process of the electrode/electrolyte interface. Figure 6 shows the Nyquist plots of MoS_2_/rGO binary composite and MoS_2_/rGO/PANI ternary composites. In high-frequency regions, the intercept of the semicircle with the X-axis represents the equivalent series resistance (R_s_) of the electrode materials, while the diameter of the semicircle corresponds to the charge transfer resistance (R_ct_) (Sk et al., 2014). From the Nyquist plots, the R_s_ of MoS_2_/rGO binary composite and MoS_2_/rGO/PANI ternary composites (MGP-50, MGP-60, MGP-70 and MGP-80) were 0.28, 0.90, 0.93, 0.97, and 1.03 Ω, respectively, indicating that the introduction of PANI could decrease the electrical conductivity of binary composite. However, R_ct_ displays a opposite trend, probablely because that the hierarchical structures of MoS_2_/rGO/PANI ternary composites show the fast charge transfer rates and reflect a preferable electrochemical performance. In low frequency regions, the nearly parallel to imaginary axis of the lines show that MoS_2_/rGO/PANI ternary composites have an ideal capacitive behavior. These results indicated that the ternary composite could improved charge storage and transportation within the electrode, which should be considered as a better electrode material (Liu et al., 2014).

## Conclusions

MoS_2_/rGO/PANI ternary composites with different amounts of PANI were successfully prepared by a two-stage synthesis combing a hydrothermal method with *in situ* chemical oxidative polymerization, and their supercapacitor performance were further investigated using electrochemical measures. The introduction of PANI in the MoS_2_/rGO binary composite not only hindered the agglomeration of MoS_2_/rGO, but also resulted in a synergistic effect among these three components. MoS_2_/rGO/PANI ternary composite with 80% PANI (MGP-80) showed the highest specific capacitance of 570 F g^−1^ at of 1 A g^−1^ which was comparatively larger than MoS_2_/rGO binary composite (200 F g^−1^). Furthermore, it was noticed that the specific capacitance of the MGP-80 composite retained more than 78.6% after 500 cycles at 1 A g^−1^. All the evidences suggest that the MoS_2_/rGO/PANI ternary composite is a high-performance electrode material for next-generation supercapacitors.

## Author contributions

The work cannot be completed without kind cooperation of all authors. L-ZB and FL: Carried out the material preparation and electrochemical test; Y-HW and S-SC: Carried out and analyzed the FT-IR, XRD, and SEM analysis; L-ZB: Wrote the paper and all authors discussed the results and revised the manuscript; Z-YZ and Y-QL: Attained the main financial support for the research and supervised all the experiments.

### Conflict of interest statement

The authors declare that the research was conducted in the absence of any commercial or financial relationships that could be construed as a potential conflict of interest.
